# LRBA Deficiency in a Patient With a Novel Homozygous Mutation Due to Chromosome 4 Segmental Uniparental Isodisomy

**DOI:** 10.3389/fimmu.2018.02397

**Published:** 2018-10-16

**Authors:** Pere Soler-Palacín, Marina Garcia-Prat, Andrea Martín-Nalda, Clara Franco-Jarava, Jacques G. Rivière, Alberto Plaja, Daniela Bezdan, Mattia Bosio, Mónica Martínez-Gallo, Stephan Ossowski, Roger Colobran

**Affiliations:** ^1^Pediatric Infectious Diseases and Immunodeficiencies Unit, Hospital Universitari Vall d'Hebron, Vall d'Hebron Research Institute, Universitat Autònoma de Barcelona, Barcelona, Spain; ^2^Jeffrey Modell Foundation Excellence Center, Barcelona, Spain; ^3^Immunology Division, Department of Cell Biology, Physiology and Immunology, Hospital Universitari Vall d'Hebron, Vall d'Hebron Research Institute, Autonomous University of Barcelona, Barcelona, Spain; ^4^Department of Clinical and Molecular Genetics, Hospital Universitari Vall d'Hebron, Barcelona, Spain; ^5^Centre for Genomic Regulation, The Barcelona Institute of Science and Technology, Barcelona, Spain; ^6^Universitat Pompeu Fabra, Barcelona, Spain; ^7^Institute of Medical Genetics and Applied Genomics, University of Tübingen, Tübingen, Germany

**Keywords:** primary immunodeficiency, LRBA deficiency, uniparental disomy, whole exome sequencing, comparative genomic hybridization array

## Abstract

LRBA deficiency was first described in 2012 as an autosomal recessive disorder caused by biallelic mutations in the *LRBA* gene (OMIM #614700). It was initially characterized as producing early-onset hypogammaglobulinemia, autoimmune manifestations, susceptibility to inflammatory bowel disease, and recurrent infection. However, further reports expanded this phenotype (including patients without hypogammaglobulinemia) and described LRBA deficiency as a clinically variable syndrome with a wide spectrum of clinical manifestations. We present the case of a female patient who presented with type 1 diabetes, psoriasis, oral thrush, and enlarged liver and spleen at the age of 8 months. She later experienced recurrent bacterial and viral infections, including pneumococcal meningitis and Epstein Barr viremia. She underwent two consecutive stem cell transplants at the age of 8 and 9 years, and ultimately died. Samples from the patient and her parents were subjected to whole exome sequencing, which revealed a homozygous 1-bp insertion in exon 23 of the patient's *LRBA* gene, resulting in frameshift and premature stop codon. The patient's healthy mother was heterozygous for the mutation and her father tested wild-type. This finding suggested that either one copy of the paternal chromosome 4 bore a deletion including the *LRBA* locus, or the patient inherited two copies of the mutant maternal *LRBA* allele. The patient's sequencing data showed a 1-Mb loss of heterozygosity region in chromosome 4, including the *LRBA* gene. Comparative genomic hybridization array of the patient's and father's genomic DNA yielded normal findings, ruling out genomic copy number abnormalities. Here, we present the first case of LRBA deficiency due to a uniparental disomy (UPD). In contrast to classical Mendelian inheritance, UPD involves inheritance of 2 copies of a chromosomal region from only 1 parent. Specifically, our patient carried a small segmental isodisomy of maternal origin affecting 1 Mb of chromosome 4.

## Introduction

Primary immunodeficiencies (PIDs) comprise a range of genetically determined diseases characterized by partial or complete loss of immune system function. Patients with PIDs typically have increased susceptibility to infections and many show evidence of immune dysregulation, manifesting as autoimmunity, allergy, or malignancy (1). In most cases, PIDs are inherited as monogenic disorders, although common variable immunodeficiency (CVID) is a clear exception. CVID is a primary antibody deficiency characterized by hypogammaglobulinaemia and increased susceptibility to infections, particularly of the respiratory tract. CVID shows considerable phenotypic and genetic heterogeneity, and monogenic forms account for < 20% of all cases (2). In the last few years, next-generation sequencing (NGS) technology has accelerated the discovery of autosomal recessive and dominant genes causing various conditions previously included in the clinical diagnosis of CVID (3, 4).

One such condition is LPS-responsive beige-like anchor (LRBA) protein deficiency (OMIM #614700). LRBA deficiency was first described in 2012 and initially characterized as producing early-onset hypogammaglobulinemia, autoimmune manifestations, susceptibility to inflammatory bowel disease, and recurrent infection (5). However, later reports expanded this phenotype to include patients without hypogammaglobulinaemia and described LRBA deficiency as a clinically variable syndrome with a wide spectrum of manifestations (6, 7).

LRBA is a cytosolic protein located in the endoplasmic reticulum, trans-Golgi apparatus, endocytosis vesicles, and lysosomes. It is ubiquitously expressed by almost all cell types, but shows higher expression levels in immune effector cells. LRBA participates in polarized vesicle trafficking and is probably involved in other critical cellular processes (5).

LRBA deficiency is an autosomal recessive disorder caused by biallelic mutations in the *LRBA* gene. Concordantly, all reported patients with a molecular diagnosis of this condition carry homozygous or compound heterozygous mutations. These mutations are distributed throughout the gene and essentially include missense mutations, splice site mutations, small indels, and nonsense mutations (6). Large structural rearrangements affecting several exons of *LRBA* have also been described in a few patients (7, 8).

Here, we present the case of a patient with LRBA deficiency due to a uniparental disomy (UPD), the first such case described. In contrast to classical Mendelian inheritance, UPD involves inheritance of two copies of a region of a chromosome from only one parent and can be heterodisomic (inheritance of a pair of homologous chromosomes from one parent) or isodisomic (inheritance of two copies of a single chromosome homolog from one parent). UPD can affect the entire chromosome or only a segment (this latter situation is termed partial or segmental UPD). The case reported here involves a cytogenetically normal female with a lethal LRBA deficiency resulting from a novel homozygous mutation. We demonstrated that the homozygosity was the result of a small segmental isodisomy of maternal origin affecting 1 Mb of chromosome 4.

## Materials and methods

### Patient

Clinical data were obtained from the patient's medical records. Written informed consent was provided by the patient's parents for the studies reported here and for the publication of this case report, in accordance with the procedures of the Ethics Review Board of Hospital Universitari Vall d'Hebron, Barcelona (Spain).

### Whole exome sequencing

#### Sample preparation and sequencing

DNA from the parent-child trio underwent whole exome sequencing (WES) as described previously (9, 10). DNA from blood was sheared using Covaris with the following settings: 300–400 bp (target size), C = 35 ng/μL, V = 45 μL, DC = 10, I = 5, C/b = 200, *t* = 040 s, water level = 12.5. WES libraries were prepared using the Illumina TruSeq DNA kit (Illumina, San Diego, CA, USA), NEXTflex Rapid DNA-Seq Kit, and NEXTflex Rapid Pre-Capture Combo Kit (BIOO Scientific, Austin, TX, USA) following the supplier's instructions. Pools of 6 samples were subjected to whole exome capture, applying Nimblegene SeqCap EZ Human Exome, version 3 (Roche NimbleGen, Madison, WI, USA). Libraries were sequenced to an average coverage of 50 × on an Illumina HiSeq 2500 sequencer using the 2 × 100 bp paired-end read protocol.

#### Computational analysis

We used the eDiVA pipeline (http://ediva.crg.eu) to analyse the sequencing data of the parent-child trio. eDiVA performs read alignment using bwa-mem followed by GATK indel realignment, variant prediction by GATK HaplotypeCaller, as well as variant annotation and variant pathogenicity classification using customized methods. Finally, causal variant prioritization was performed according to four inheritance patterns (autosomal recessive homozygous, autosomal recessive compound heterozygous, autosomal dominant *de novo*, X-linked), as described (9, 10). Furthermore, we identified regions affected by loss of heterozygosity using Bcfttols v1.3 (11), and detected copy number variants using Conifer (12).

### Microarray-based comparative genomic hybridization

DNA copy-number variations were investigated with an array comparative genomic hybridization assay using the CytoSure Constitutional v3 array 8 × 60 K (Oxford Gene Technology, UK) according to manufacturer recommendations on DNA extracted from peripheral blood with the GENTRA Puregene Kit (Qiagen, Hilden, Germany). DNA from the patient and her father were compared with a reference DNA sample (healthy control). Data analysis was performed by Cytogenomics 2.1 software with the ADM-2 algorithm and a minimum of three consecutive probes to detect an anomaly.

## Results

The patient was a Caucasian girl with clinical onset at 8 months of age. She was the only daughter of a non-consanguineous couple; her mother had psoriasis and her father vitiligo. Her grandfather was affected with type 1 insulin-dependent diabetes mellitus, diagnosed at the age of 30. Her mother had no previous miscarriages and the prenatal history was uneventful. No allergies were demonstrated and the vaccination schedule, including live vaccines, had been updated to the age of 12 months with no significant adverse events.

At the age of 8 months the patient presented with type 1 diabetes, psoriasis, oral thrush, and enlarged liver and spleen. Two months later, lower right lobe pneumonia caused by *Streptococcus pneumoniae* was diagnosed. At that point, splenomegaly persisted and Epstein Barr virus (EBV) viremia was detected (viral load 9,298 copies/mL). When she was 2 years old, pneumococcal meningitis and immune thrombocytopenia were diagnosed and she experienced recurrent pneumonia despite immunoglobulin replacement therapy and antibiotic prophylaxis. These episodes led to the development of bronchiectasis in the left lower lobe and lingula, documented on CT scanning when she was 4 years old. Immunological studies performed at that time showed essentially normal lymphocyte counts, although T cells with effector phenotype were expanded and B and NK cell numbers were slightly decreased (Table 1). Despite low/normal immunoglobulin values, specific antibody production was impaired. NK cytotoxicity was repeatedly normal. When she was 28 months old, an EBV-related lymphoproliferative syndrome (mature B cell lymphoma) was diagnosed and appropriate chemotherapy was started with a good clinical and radiological response. Subsequently, she had varicella zoster meningitis at the age of 3 and severe inflammatory enteropathy starting at the age of 5.

**Table 1:** Immunological parameters.

**Parameter**	**Patient values**	**Reference Values**
Leucocytes, ×10^9^/L	5.8	5.5–18
Lymphocytes, % | ×10^9^/L	65 | **3.8**	52–72 | 1.2–3.4
CD3+, % | ×10^9^/L	**83** | 3.1	54–76 | 1.6–6.7
CD3+CD4+, % | ×10^9^/L	52 | 2	31–54 | 1–4.6
CD3+CD8+, % | ×10^9^/L	28 | 1.1	12–28 | 0.4–2.1
CD19+, % | ×10^9^/L	**11 | 0.4**	15–39 | 0.6–2.7
CD56+CD3−, % | ×10^9^/L	4 | **0.16**	3–17 | 0.2–1.2
CD3+CD4+HLA–DR+, %	8	2–8
CD3+CD8+HLA–DR+, %	**19**	2–8
CD4+CD45RO+, %	**63**	23–34
CD8+CD45RO+, %	**77**	25–37
Total memory B cells, %	13.6	>13% from total B cells
Switch memory B cells, %	**4.7**	>6% from total B cells
Lymphocyte proliferation response to PHA	Normal	-
Lymphocyte proliferation response to anti–CD3	Normal	-
TCR repertoire CD4+	Polyclonal	Polyclonal pattern
TCR repertoire CD8+	**Oligoclonal**	Polyclonal pattern
TRECs/100 ng gDNA	**0**	18–57
NK cytoto×icity	Normal	-
IgG, mg/dL	399	360–1236
IgA, mg/dL	**<10**	11–153
IgM, mg/dL	**<10**	48–245
C3, mg/dL	136	85–180
C4, mg/dL	**43**	10–40
sCD25, IU/mL	**191**	25–124 (fHLH cut off: 2400)
Coomb's test	Positive	-
CMV IgG	Positive	-
CMV IgM	Negative	-
EBV IgG	Positive	-
EBV IgM	Negative	-

*The most significant altered values are marked in bold*.

The patient underwent two consecutive stem cell transplants (SCT) due to loss of the first graft, at the age of 7 and 8 years, respectively, to treat persistent, refractory enteropathy and bone marrow failure. One month after the second transplant, she was admitted to the intensive care unit because of respiratory distress and sadly, she died. Disseminated toxoplasmosis was demonstrated in post-mortem studies.

**Figure 1:**
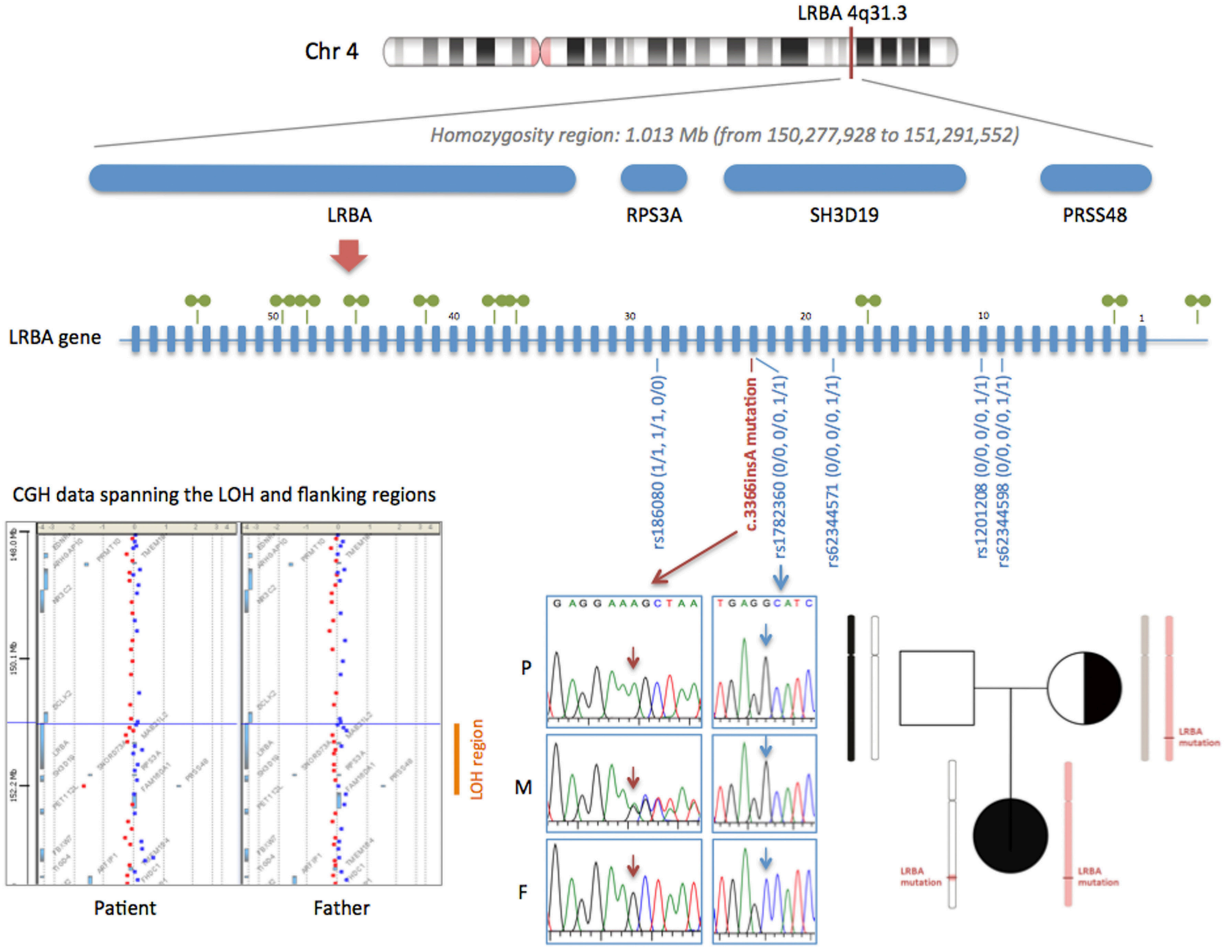
Segmental isodisomy of maternal origin containing the c.3366insA *LRBA* mutation. The LOH region in chromosome 4 of the patient is indicated by a red line. The LOH region, detected by analyzing WES data, includes four genes (LRBA, RPS3A, SH3D19, and PRSS48). A diagram of the *LRBA* gene with its 58 exons is shown. The 10 CGH array probes located along the *LRBA* gene are depicted with green symbols. All *LRBA* probes displayed a normal signal, confirming a copy number-neutral region. The c.3366insA mutation is shown in red (electropherograms of the patient and her parents). Five common SNPs surrounding the *LRBA* mutation are also shown in blue. The genotype of the patient (P) and her mother (M) and father (F) are indicated using the numbers 0 and 1 for the 2 possible alleles. Array-CGH data spanning the LOH and flanking regions is shown. gDNA from patient and father was labeled with Cy3 (red) and control gDNA with Cy5 (blue). The pedigree of the family shows the inherited chromosome 4, with the segmental isodisomy of maternal origin.

Whole exome sequencing revealed a homozygous 1-bp insertion in exon 23 of the patient's *LRBA* gene, causing a frameshift and introduction of a premature stop codon resulting in a predicted truncated protein of 1,123 aa (wild-type LRBA protein: 2,863 aa). This mutation had not been reported in the literature or databases, and, following the recommendations of the Human Genome Variation Society (HGVS), we named it c.3366insA (p.Ala1123SerfsX1). WES data from the parents showed that the patient's healthy mother was heterozygous for the mutation and her father did not carry the mutation. Considering the autosomal recessive inheritance of LRBA deficiency, this was not consistent with the homozygous occurrence in our patient. We confirmed the genotypes by Sanger sequencing (Figure 1), and paternity was confirmed by high-resolution HLA typing. These findings suggested that either the paternal chromosome 4 bore a deletion including the *LRBA* locus (at least exon 23), or the patient inherited two copies of the mutant maternal *LRBA* allele. The patient's WES data showed a loss of heterozygosity (LOH) region of 1.013 Mb in chromosome 4 including the *LRBA* gene (Figure 1). LOH regions can be copy number-neutral or show copy number loss. Comparative genomic hybridization (CGH) array and WES-based copy number analysis of the patient's and father's genomic DNA yielded normal results, thereby ruling out genomic copy number abnormalities. We carefully checked the 10 CGH array probes located along the *LRBA* gene, which displayed a normal signal (Figure 1). In this 1.013-Mb region, the patient was homozygous for a haplotype present in the mother. Specifically, within the LOH region we detected 5 common single-nucleotide polymorphisms (SNPs) surrounding the *LRBA* mutation, in which the patient had not inherited any allele from her father. The genotypes of these SNPs were confirmed by Sanger sequencing (Figure 1). Collectively, these results indicate that the homozygous state of the patient's c.3366insA *LRBA* mutation was due to a segmental maternal isodisomy (Figure 1).

## Discussion

Mutations in the *LRBA* gene encoding the LRBA protein are associated with an autosomal recessive monogenic disorder called LRBA deficiency and Autoimmunity, regulatory T (Treg) cell defects, Autoimmune Infiltration and Enteropathy (LATAIE) syndrome or, more commonly, merely LRBA deficiency (5). Why LRBA deficiency leads to this immune dysregulation syndrome was elucidated in 2015 when a study demonstrated that LRBA interacts with the cytoplasmic tail of CTLA4 and protects it from being sorted to lysosomes for degradation (13). Therefore, LRBA deficiency triggers a de facto CTLA4 deficiency. Not surprisingly, deleterious heterozygous mutations in the CTLA4 gene cause an autosomal dominant disorder called CTLA-4 Haploinsufficiency with Autoimmune Infiltration (CHAI) (or simply CTLA4 deficiency) which has similar symptoms (14, 15). Remarkably, LRBA deficiency results in lower CTLA4 levels than those of CTLA4-deficient patients, explaining the earlier disease onset, the nearly complete penetrance, and the greater severity of LATAIE compared to CHAI disease (16).

More than 70 patients with LRBA deficiency have been reported to date, with a range of diverse mutations identified and with highly variable clinical and immunologic characteristics including chronic diarrhea, hypogammaglobulinaemia, autoimmune disorders, organomegaly, recurrent infection, and combinations of these phenotypes (5–7). From the clinical viewpoint, our patient presented a severe LRBA-deficient phenotype when compared to patients in previous studies (6). She showed immune dysregulation, organomegaly, recurrent infection, and failure to thrive, thus fulfilling nearly the entire clinical spectrum of the disease. Due to immune dysregulation, the patient was treated with steroids and rapamycin but the clinical response was suboptimal and she required HSCT, which has been proposed as an appropriate treatment option (17, 18), but unfortunately, she died. Of note, and similar to what has been seen in previous cases, our patient had a lymphoid malignancy, suggesting that proliferative disease may be another feature of LRBA deficiency (5, 13, 19).

Using massive parallel sequencing (WES) we found a homozygous loss of function mutation in the *LRBA* gene, c.3366insA (p.Ala1123SerfsX1), which perfectly fit the patient's clinical phenotype. In the analysis of WES data, no additional PID related mutations were found. The most interesting part of this study is the uncommon genetic mechanism of heredity that led to this homozygous mutation (considering that the father did not carry the mutation). By combining data from a CGH array with WES computational analysis, we found a copy number-neutral LOH region of 1 Mb in chromosome 4 including the *LRBA* gene. These results, together with the marker segregation pattern, indicate that the homozygous state of the patient's c.3366insA *LRBA* mutation was due to segmental maternal isodisomy.

Uniparental disomy is a condition in which both copies of an entire chromosome or a specific chromosomal region are inherited from the same progenitor. UPD can be pathogenic, because of either imprinting defects or inheritance of a recessive condition from a single carrier parent (20). Specifically, our patient had segmental isodisomy of maternal origin, which means that she inherited 2 copies of a fragment (~1 Mb) of a single chromosome 4 from her mother. Segmental isodisomy is considered the result of post-zygotic mitotic recombination after normal fertilization (21), a well-recognized major mechanism of the LOH of tumor suppressor genes in cancer (22). Segmental UPD is a fairly common event, found in up to 15% of control populations (23). Usually, segmental UDPs have a relatively small size (median, 8.7 Mb), are primarily interstitial (22), and typically do not exert any adverse effect on the individual. The probability that a segmental UPD reveals an autosomal recessive disease is very low. Consequently, UPD as the cause of a PID is extremely rare.

To our knowledge, only 10 cases of PID due to UPD (including the patient described here) have been reported to date. We have summarized them Table 2. Nine of the 10 cases have been reported in the last 10 years. This is not surprising, as recent technological advances, such as SNP arrays and NGS, have facilitated detection of UPD. All reported cases are isodisomies or mixed forms, according to the recessive inheritance of the diseases, and 7 of the 10 involve the entire chromosome. Our study reports the smallest segmental UPD described in PIDs, with a fragment of only 1 Mb. Most UPDs in PIDs are of maternal origin, which concurs with the observation that maternal UPD is 1.6 to 3-fold more common than paternal UPD of an entire chromosome (33).

**Table 2:** Reported cases of uniparental disomy in primary immunodeficiencies.

**Gene**	**Chr**	**Mutation**	**Isodisomy/Heterodisomy**	**Size**	**Origin**	**Year**	**References**
LYST	1	c.2620delT/Truncated protein	Isodisomy	Whole chromosome	Maternal	1999	(24)
PRF1	10	c.902C>A/p.Ser301X	Mixed form	Whole chromosome	Maternal	2008	(25)
IFNGR1	6	p.Tyr113X	Isodisomy	Whole chromosome	Maternal	2010	(26)
CD45	1	c.1618A>T/p.Lys540X	Isodisomy	Whole chromosome	Maternal	2012	(27)
LCK	1	c.1022T>C/p.Leu341Pro	Isodisomy	Whole chromosome	Maternal	2012	(28)
ADA	20	c.956_960del/Truncated protein	Mixed form	Whole chromosome	Maternal	2013	(29)
CARD9	9	c.52C>T/p.Arg18Trp	Isodisomy	Segmental (33 Mb)	Maternal	2015	(30)
CYBA	16	c.354 C>A/p.Ser118Arg	Isodisomy	Segmental (38 Mb)	Maternal	2017	(31)
IRF4	6	c.1213-2A>G/p.V405GfsTer127	Isodisomy	Whole chromosome	Maternal	2018	(32)
LRBA	4	c.3366insA/Truncated protein	Isodisomy	Segmental (1 Mb)	Maternal	2018	This study

A final relevant aspect of this case are the consequences in terms of genetic counseling: as segmental UPD is basically a rare, accidental mitotic error, the recurrence risk for siblings is not 25% as in general autosomal recessive diseases, but can be considered negligible or, at the least, much lower.

## Author contributions

PS-P was the principal clinician in charge of the patient's care and wrote part of the manuscript. MG-P, CF-J, and MM-G performed the immunological analysis. AM-N and JR were involved in management of the patient. AP performed the comparative genomic hybridization array and data analysis. DB, MB, and SO performed whole exome sequencing and the data analysis. RC performed the genetic analysis and was responsible for designing the study, writing the manuscript, and approving the final draft. All authors reviewed the manuscript and contributed to the final draft.

### Conflict of interest statement

The authors declare that the research was conducted in the absence of any commercial or financial relationships that could be construed as a potential conflict of interest.
